# Insulin Bolus Patterns in Newly Diagnosed Youth With Type 1 Diabetes Using a Hybrid Closed-Loop Insulin Delivery System

**DOI:** 10.1177/19322968251409790

**Published:** 2026-01-22

**Authors:** Chloë Royston, Julia Ware, Janet M. Allen, Malgorzata E. Wilinska, Sara Hartnell, Ajay Thankamony, Tabitha Randell, Atrayee Ghatak, Rachel E. J. Besser, Daniela Elleri, Nicola Trevelyan, Fiona M. Campbell, Roman Hovorka, Charlotte K. Boughton, CLOuD Consortium

**Affiliations:** 1 Institute of Metabolic Science-Metabolic Research Laboratories and Medical Research Council Metabolic Diseases Unit, University of Cambridge, Cambridge, UK; 2 Department of Paediatrics, University of Cambridge, Cambridge, UK; 3 Wolfson Diabetes and Endocrine Clinic, Cambridge University Hospitals National Health Service Foundation Trust, Cambridge, UK; 4 Department of Paediatric Diabetes and Endocrinology, Nottingham Children’s Hospital, Nottingham, UK; 5 Department of Diabetes, Alder Hey Children’s National Health Service Foundation Trust, Liverpool, UK; 6 Department of Paediatrics, University of Oxford, Oxford, UK; 7 National Institute for Health and Care Research Oxford Biomedical Research Centre, John Radcliffe Hospital, Oxford, UK; 8 Department of Diabetes, Royal Hospital for Sick Children, Edinburgh, UK; 9 Paediatric Diabetes, Southampton Children’s Hospital, Southampton, UK; 10 Department of Paediatric Diabetes, Leeds Children’s Hospital, Leeds, UK

**Keywords:** bolus patterns, hybrid closed-loop, newly diagnosed, type 1 diabetes

## Abstract

**Background:**

This study aimed to investigate the decline over time in the proportion of total daily insulin delivered as boluses in newly diagnosed youth with type 1 diabetes using a hybrid closed-loop system.

**Method:**

A secondary analysis was conducted using data from the CLOuD study, an open-label, multicenter, randomized, parallel hybrid closed-loop trial to investigate bolus patterns in youth with newly diagnosed type 1 diabetes.

**Results:**

Over the 48-month trial period, the proportion of total daily insulin delivered as carbohydrate-related boluses decreased from 58% to 34%. There was a decreasing trend in the median (interquartile range) amount of carbohydrates entered per day from 236 (204, 253) g to 184 (127, 232) g, and the number of carbohydrate-related boluses per day from 5.5 (4.6, 6.5) to 3.7 (2.9, 5.2) over the 48 months. Mean ± SD daily carbohydrate-related bolus insulin increased from 15.1 ± 6.6 to 22.0 ± 9.0 units/d, and the amount of insulin delivered per 10 g of carbohydrate more than doubled from 0.6 (0.5, 0.8) units to 1.3 (0.9, 1.5) units. The postprandial change in glucose (measured as the difference between peak glucose 30 to 180 minutes post carbohydrate-related bolus and glucose on carbohydrate-related bolus delivery) changed from 49 (45, 54) to 59 (53, 66) mg/dL.

**Conclusions:**

The decline in the proportion of total daily insulin delivered for as bolus is likely attributable to a combination of missed boluses and under-bolusing, while the closed-loop algorithm compensates for the missed or insufficient carbohydrate-related insulin delivery by increasing basal insulin delivery.

## Introduction

Type 1 diabetes is a chronic autoimmune condition characterized by insulin deficiency resulting from immune-mediated β-cell destruction. Following type 1 diabetes diagnosis endogenous insulin production declines over time leading to increased exogenous insulin requirements.^1,2^ In a previous analysis of hybrid closed-loop insulin delivery in youth newly diagnosed with type 1 diabetes for a period of four years follow-up, we observed an increasing proportion of closed-loop insulin delivered as basal rather than bolus insulin. The factors underlying the decline in the proportion of total daily insulin delivered as bolus have not yet been investigated.

Mealtime bolusing is reported as a challenge of diabetes management in adolescents and missed mealtime boluses is frequently cited as a contributing factor to suboptimal glucose outcomes in this age group.^3,4^ We aimed to assess whether missed boluses or other factors could explain the reducing proportion of insulin delivered as boluses over time in this cohort.

The aim of this post hoc analysis was to evaluate bolus insulin patterns in newly diagnosed youth with type 1 diabetes using a hybrid closed-loop system, and to explore why the proportion of total daily insulin delivered as boluses decreases over time.

## Methods

### Study Design and Population

A secondary analysis of an open-label, multicenter, randomized, parallel hybrid closed-loop trial (CLOuD trial) was undertaken to assess bolus insulin delivery patterns in the first 48 months following type 1 diabetes diagnosis. Participants aged ≥10 and <17 years were recruited within 21 days of diagnosis of type 1 diabetes and were randomized to either hybrid closed-loop or standard insulin therapy for 24 months. Participants were offered a 24-month optional extension phase with the allocated treatment. Participants aged 16 years and parents/guardians of participants <16 years opting to continue in the extension phase were asked to reconsent. Written assent was obtained from participants <16 years. The present analysis includes only participants who used hybrid closed-loop therapy. The study was approved by an independent research ethics committee.

### Closed-Loop System

The Cambridge model predictive control algorithm (version 0.3.71; CamDiab Ltd, Cambridge, UK) was implemented in two hardware configurations sequentially. The FlorenceM configuration consisted of an unlocked Android smartphone running the algorithm which communicated with a modified, next-generation sensor-augmented 640G Medtronic insulin pump (Medtronic MiniMed, California) through a proprietary translater, and a Medtronic continuous glucose monitor (CGM) transmitter with Guardian 3 sensor. The CamAPS FX configuration used an unlocked Android smartphone to house the CamAPS FX closed-loop app, a Dana Diabecare RS insulin pump (Sooil Development, Seoul, Korea) or YpsoPump (Ypsomed, Burgdorf, Switzerland), and either the Dexcom G6 (Dexcom, San Diego, California) or FreeStyle Libre 3 (Abbott Diabetes Care, Alameda, California) CGM.

Both configurations used an algorithm housed in a mobile app to automatically adjust the insulin infusion rate every 8 to 12 minutes. All insulin delivered by the algorithm was considered basal insulin. The system was initialized by entering the user’s weight and total daily dose, while insulin sensitivity and active insulin time were automatically calculated and adjusted as necessary. The algorithm used adaptive learning with respect to total daily insulin dose, diurnal variation, and insulin delivery around meals. Insulin pump basal rates were preset by the user and only used if the system was not in automode. These do not influence the algorithm insulin delivery. The insulin to carbohydrate ratio (ICR) was entered into the system at set-up and used by the algorithm for meal bolus calculations. All “non-carbohydrate”-related insulin delivery was manually administered by the user.

### Data Analysis

Metrics were calculated daily for each participant and then summarized over three-month (91‑day) periods for the first 24 months and six-month (182-day) periods for the second 24 months. Time periods were calculated from the day after diagnosis. All metrics were summarized with equal weighting across participants. To be included in the analysis, data were only considered if participants spent at least 50% of each 24-hour period in closed-loop mode. Additionally, a minimum of 30 days of data were required for inclusion in each 91-day period and at least 60 days for each 182-day period.

Metrics included total daily carbohydrate-related bolus insulin (defined as a bolus delivered within 5 minutes of a carbohydrate entry), the proportion of total insulin delivered as carbohydrate-related boluses, number of carbohydrate-related boluses per day, total daily carbohydrates entered per day, units of insulin delivered per 10 g of carbohydrate (ICR), postprandial change in glucose, and time in target glucose range between 70 and 180 mg/dL. Insulin delivered per 10 g of carbohydrate was calculated by dividing the total bolus insulin delivered during each 91- or 182-day period by the total amount of carbohydrate entered during that same period and multiplying that by 10. Based on typical timing of postprandial glucose excursions, postprandial change in glucose was defined as the difference between the peak glucose level (found within 30 to 180 minutes after a carbohydrate-related bolus) and the glucose at the time of the carbohydrate-related insulin bolus delivery.^5^

Data analysis was completed using R Studio (R Foundation for Statistical Computing, Vienna, Austria). Data are presented as median (interquartile range, IQR) or mean ± standard deviation (SD).

## Results

**Figure 1.**
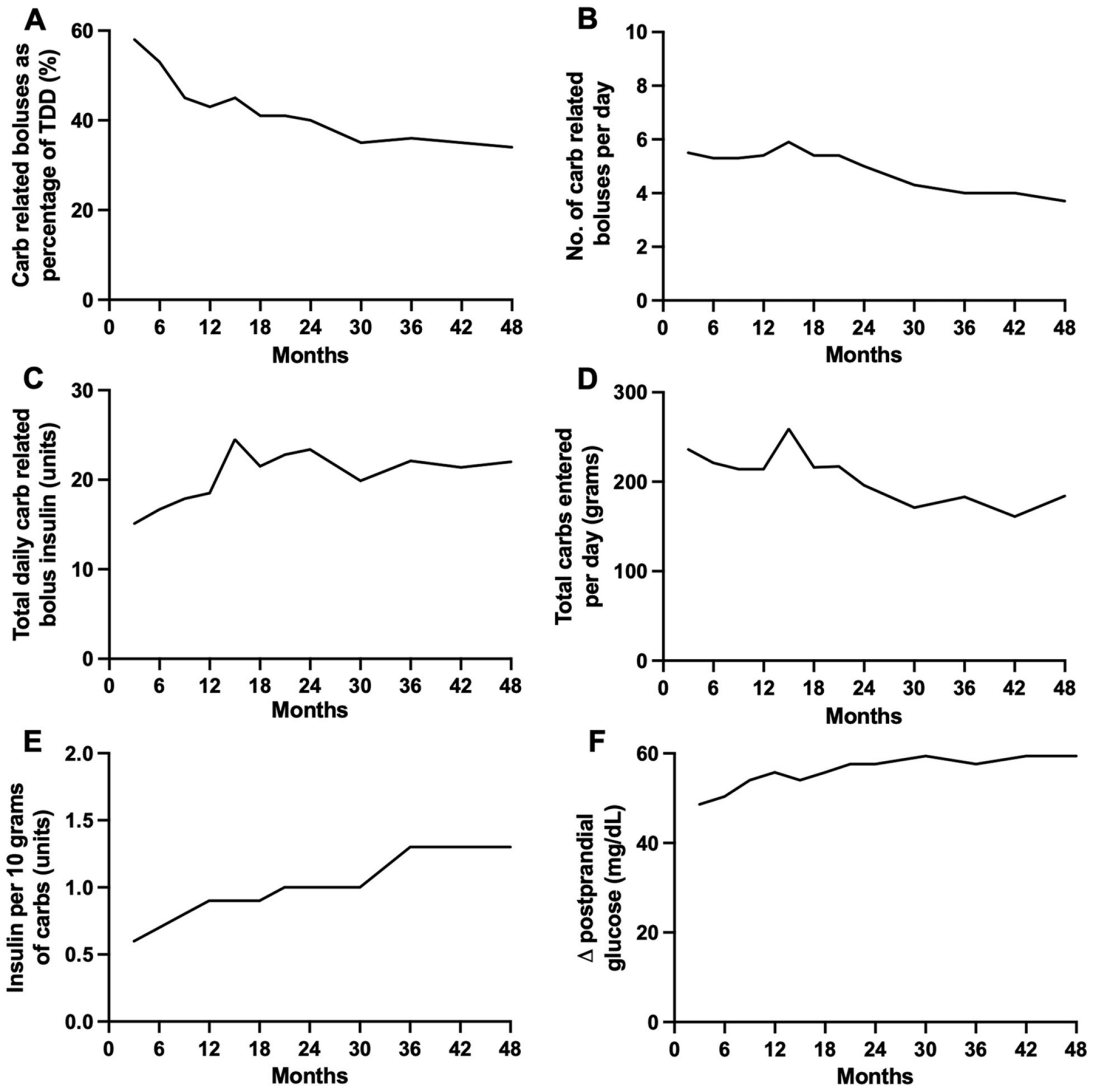
Trends in carbohydrate-related insulin boluses and glycemic outcomes during hybrid closed-loop insulin delivery over the 48 months following diagnosis of type 1 diabetes: (a) Proportion of total daily insulin as carbohydrate-related bolus insulin (%; mean). (b) Carbohydrate-related bolus frequency (per day; median). (c) Total daily carbohydrate-related bolus (units; mean). (d) Total carbohydrate entered per day (g; median). (e) Insulin delivered per 10 g of carbohydrate (median). (f) Change in postprandial glucose calculated as difference between peak glucose 30 to 180 minutes post carbohydrate-related bolus and glucose on carbohydrate-related bolus delivery (mg/dL; median). Abbreviations: Carb(s), carbohydrate(s); TDD, total daily dose.

A total of 48 participants (23 female and 25 male) aged 10 to 16.9 years were included in the analysis (Table 1). One participant was excluded as an outlier due to behavioral (deliberate carbohydrate restriction) factors impacting on insulin requirements. The trends in carbohydrate-related insulin boluses and glycemic outcomes are shown in Figure 1.

**Table 1.** Demographics.

Characteristics	Overall (N = 48)	Female (N = 23)	Male (N = 25)
Mean age at enrolment, y	12 ± 2	12 ± 2	12 ± 2
BMI percentile	19 ± 3	19 ± 4	19 ± 3
Race—No. (%)			
White	41 (85)	17 (74)	24 (96)
Black	1 (2)	1 (4)	0 (0)
Asian	2 (4)	2 (9)	0 (0)
More than one race	4 (8)	3 (13)	1 (4)
Glycated hemoglobin level, %	10.7 ± 1.8	10.8 ± 2.0	10.6 ± 1.6
Diabetic ketoacidosis at diagnosis, No. (%)	15 (31)	9 (39)	6 (24)

Data presented as mean ± standard deviation.

### Bolus Insulin Delivery

The proportion of total daily dose of insulin accounted for by carbohydrate-related insulin boluses declined from 58% at zero to three months to 34% at 42 to 48 months (Table 2, Figure 1a). The median (IQR) number of carbohydrate-related boluses administered per day decreased from 5.5 (4.6, 6.5) in the first three months to 3.7 (2.9, 5.2) in the final three months (Table 2, Figure 1b).

**Table 2.** Bolus Patterns and Glycemic Outcomes.

Months since diagnosis	0-3 months	3-6 months	6-9 months	9-12 months	12-15 months	15-18 months	18-21 months	21-24 months	24-30 months	30-36 months	36-42 months	42-48 months
Number of participants	29	38	31	35	42	41	38	41	37	39	38	37
Total daily carb-related insulin bolus (units/d)	15.1 ± 6.6	16.7 ± 8.9	17.9 ± 10.4	18.5 ± 9.6	24.5 ± 17.1	21.5 ± 13.0	22.8 ± 12.0	23.4 ± 14.3	19.9 ± 8.6	22.1 ± 10.1	21.4 ± 8.8	22.0 ± 9.0
Total daily non-carb-related insulin bolus (units/d)	0.3 ± 0.6	0.9 ± 2.6	1.4 ± 2.9	1.2 ± 1.9	1.6 ± 1.7	2.1 ± 2.5	2.4 ± 2.8	1.7 ± 1.8	2.2 ± 2.5	2.3 ± 2.8	2.5 ± 2.7	3.3 ± 4.0
Carb-related bolus percentage of TDD, %	58 ± 9	53 ± 10	45 ± 11	43 ± 11	45 ± 14	41 ± 14	41 ± 13	40 ± 13	35 ± 12	36 ± 13	35 ± 12	34 ± 12
No. of carb-related boluses per day	5.5 (4.6, 6.5)	5.3 (4.4, 6.9)	5.3 (4.3, 6.6)	5.4 (4.5, 6.0)	5.9 (4.9, 8.0)	5.4 (4.2, 6.8)	5.4 (4.0, 6.5)	5.0 (3.4, 6.6)	4.3 (3.3, 5.2)	4.0 (3.0, 5.5)	4.0 (2.8, 5.1)	3.7 (2.9, 5.2)
Total daily carb entry, g	236 (204, 253)	221 (185, 248)	214 (182, 257)	214 (187, 250)	259 (193, 326)	216 (177, 279)	217 (172, 264)	196 (161, 238)	171 (137, 232)	183 (136, 223)	161 (123, 216)	184 (127, 232)
Insulin ratio per 10 g carb (units)	0.6 (0.5, 0.8)	0.7 (0.5, 0.9)	0.8 (0.5, 0.9)	0.9 (0.5, 1.0)	0.9 (0.6, 1.1)	0.9 (0.6, 1.2)	1.0 (0.7, 1.3)	1.0 (0.8, 1.3)	1.0 (0.8, 1.3)	1.3 (0.9, 1.5)	1.3 (1.0, 1.6)	1.3 (0.9, 1.5)
Δ postprandial glucose, mg/dL^a^	49 (45, 54)	50 (44, 59)	54 (49, 63)	55 (49, 60)	54 (47, 69)	56 (51, 66)	57 (53, 63)	57 (50, 63)	59 (54, 65)	58 (52, 63)	59 (51, 65)	59 (53, 66)
TIR (70-180 mg/dL), %	81 ± 13	81 ± 9	74 ± 10	75 ± 10	70 ± 13	71 ± 10	71 ± 10	72 ± 13	69 ± 10	68 ± 10	68 ± 10	65 ± 13

Data presented as mean ± standard deviation or median (interquartile range).

Abbreviations: TDD, total daily dose; TIR, time in range between 70 and 180 mg/dL; carb, carbohydrate.

a Calculated as difference between peak glucose 30 to 180 minutes post carb-related bolus and glucose on carb-related bolus delivery.

The absolute number of units of insulin delivered for carbohydrate intake per day increased by approximately 1.5-fold from 15.1 ± 6.6 units/d at zero to three months to 22.0 ± 9.0 units/d at 4248 months. This increase occurred most rapidly during the first 15 months, rising from 15.1 ± 6.6 to 24.5 ± 17.1 units/d (Table 2, Figure 1c). Non-carbohydrate-related bolus delivery was low overall but also increased from 0.3 ± 0.6 units/d at months 0 to 3 after diagnosis to 3.3 ± 4.0 units/d during months 42 to 48 (Table 2).

### Carbohydrate Entry

Total carbohydrate entered into the closed-loop app per day decreased over the study period by approximately 22% from 236 (204, 253) g to 184 (127, 232) g (Table 2, Figure 1d).

The ratio of insulin delivered per 10 g of carbohydrate increased by just over twofold over the 48 months from 0.6 (0.5, 0.8) units in months 0 to 3 to 1.3 (0.9, 1.5) units in months 42 to 48 (Table 2, Figure 1e).

### Glucose Outcomes

Despite the increasing ratio of insulin delivered per 10 g of carbohydrate, the postprandial glucose excursion increased by approximately 20% over the 48 months from 49 (45, 54) mg/dL at 0 to 3 months to 59 (53, 66) mg/dL at 42 to 48 months (Table 2, Figure 1f). Time in target glucose range (70 to 180 mg/dL) showed a trend toward a decrease from 81 ± 13% at zero to three months to 65 ± 13% at 42-48 months (Table 2).

### Subgroup Analysis (Time in Range ≥70%)

We completed a subanalysis of individuals with a mean time in target glucose range of ≥70% (n = 30). The trends in this cohort reflect the same trends observed in the overall population in this study. However, the small sample size limits the interpretation of this finding.

## Discussion

Our analysis suggests that the decreasing proportion of total daily insulin delivered as boluses may be driven by a combination of reduced carbohydrate-related bolus frequency and inadequate adjustment of carbohydrate ratios or carbohydrate estimation.

The initial number of carbohydrate-related boluses (5.5 times per day) was comparable to a prior study analyzing bolus delivery patterns in adults with type 1 diabetes and an HbA1c <7.5% using a hybrid closed-loop system, which reported an average of 6.0 boluses per day.^6^ However, by the end of the 48-month period, our analysis showed a decrease of between one to two boluses per day, along with a decreasing time in range (TIR). Missed boluses is often cited as a key factor contributing toward suboptimal glycemic outcomes, particularly among adolescents,^7,8^ which does seem to be supported in this analysis.

The total amount of insulin delivered for carbohydrate intake each day increased despite fewer boluses. This was reflected in the 1.5-fold rise in insulin ratio per 10 g of carbohydrate, suggesting that insulin-to-carbohydrate ratios are being adjusted over time. This increasing in insulin requirements is expected immediately following a type 1 diabetes diagnosis and later due to insulin resistance often occurring during growth and puberty.^1,2,9^ The increase in postprandial glucose excursions over the 48-month study period, alongside a decreasing proportion of bolus insulin relative to total daily dose despite adjustments to insulin-to-carbohydrate ratios, suggests that these adjustments were inadequate. The insulin-to-carbohydrate ratios likely remained too weak, or carbohydrate entry is increasingly underestimated over time, potentially due to growing portion sizes in this population.

Changes in basal insulin delivery directly reflect the closed-loop algorithm adjustments. In contrast, bolus insulin delivery is user-initiated and is primarily associated with carbohydrate intake. The decreasing proportion of total daily insulin accounted for by carbohydrate-related boluses, combined with the observed trends, suggests the algorithm is delivering insulin to compensate for missed boluses or insufficient insulin delivery surrounding carbohydrate intake. The algorithm compensates for suboptimal carbohydrate-related bolusing and is likely to be delivering insulin later in response to postprandial glucose spikes rather than insulin ideally having been administered as a premeal bolus. While hybrid closed-loop systems can partially compensate for inaccurate or even missed meal boluses, this is not always sufficient to stop postprandial glucose excursions due to the delay in insulin action.

Strengths of the present analysis include the long duration of hybrid closed-loop insulin delivery following diagnosis of type 1 diabetes in youth. Limitations include the small sample size (N = 48), limited ethnic diversity (85% white), the absence of dietary records and weight measurements for participants and the lack of pubertal status and psychosocial context which may impact on the results.

## Conclusions

In conclusion, over the 48 months following type 1 diabetes diagnosis, the decline in the proportion of total daily insulin delivered as boluses is attributable to a combination of reduced carbohydrate-related bolus frequency and inadequate adjustment of carbohydrate ratios or carbohydrate estimation. The closed-loop algorithm partially compensates for missed or insufficient carbohydrate-related bolus insulin; however, additional education surrounding carbohydrate counting, meal blousing, and ICR adjustments may be beneficial.

## References

[1] DiMeglio LA Evans-Molina C Oram RA. Type 1 diabetes. Lancet. 2018;391(10138):2449-2462. 29916386 10.1016/S0140-6736(18)31320-5 PMC6661119

[2] Ware J Boughton CK Allen JM , et al. Effect of 48 months of closed-loop insulin delivery on residual C-peptide secretion and glycemic control in newly diagnosed youth with type 1 diabetes: a randomized trial. Diabetes Care. 2024;47(8):1441-1448. 38924772 10.2337/dc24-0360 PMC11272979

[3] Datye KA Moore DJ Russell WE Jaser SS. A review of adolescent adherence in type 1 diabetes and the untapped potential of diabetes providers to improve outcomes. Curr Diab Rep. 2015;15(8):51. 26084580 10.1007/s11892-015-0621-6 PMC4692366

[4] Morrissey EC Dinneen SF Lowry M de Koning EJ Kunneman M. Reimagining care for young adults living with type 1 diabetes. J Diabetes Investig. 2022;13(8):1294-1299. 10.1111/jdi.13824 PMC9340877 35511075

[5] Daenen S Sola-Gazagnes A M’Bemba J , et al. Peak-time determination of post-meal glucose excursions in insulin-treated diabetic patients. Diabetes Metab. 2010;36(2):165-169. 20226708 10.1016/j.diabet.2009.12.002

[6] Bally L Thabit H Ruan Y , et al. Bolusing frequency and amount impacts glucose control during hybrid closed-loop. Diabet Med. 2018;35(3):347-351. 28755444 10.1111/dme.13436 PMC5788742

[7] Hood KK Peterson CM Rohan JM Drotar D. Association between adherence and glycemic control in pediatric type 1 diabetes: a meta-analysis. Pediatrics. 2009;124(6):e1171-e1179. 10.1542/peds.2009-0207 19884476

[8] Westen SC Warnick JL Albanese-O’Neill A , et al. Objectively measured adherence in adolescents with type 1 diabetes on multiple daily injections and insulin pump therapy. J Pediatr Psychol. 2019;44(1):21-31. 30184209 10.1093/jpepsy/jsy064

[9] Amiel SA Sherwin RS Simonson DC Lauritano AA Tamborlane WV. Impaired insulin action in puberty. A contributing factor to poor glycemic control in adolescents with diabetes. N Engl J Med. 1986;315(4):215-219. 3523245 10.1056/NEJM198607243150402

